# States May Regulate Medical Practice

**Published:** 1889-05

**Authors:** 


### States May Regulate Medical Practice.

An interesting decision has been rendered by the Supreme Court of the United States, supporting the right claimed by the State Board of Health of West Virginia to make rules for the regulation of the practice of medicine. The statutes of that State enact that every practitioner must qualify himself for legal practice by obtaining from the Board a certificate that he is a graduate of a reputable medical college or has been ten years in the State engaged in practice, or that he has passed a satisfactory examination before the Board. A practitioner, named F. M. Dent, having been convicted of illegal practice, carried the case up to the State Court of Appeals, and thence to the United States Supreme Court, asserting that the law was unconstitutional, inasmuch as it deprived him of liberty and property without due process of law.

The decision of the Supreme Court says: "The power of the State to provide for the general welfare of its people authorizes it to prescribe all such regulations as may be necessary to secure the people against the consequences of ignorance and incapacity as well as of deception and fraud. One means to secure this end is the method adopted by the State of West Virginia. If the means adopted are appropriate to the calling or profession, and obtainable by reasonable study or application, no objection to their validity can be raised."

If it is the right of the State under this weighty and memorable decision, to protect its citizens from pretenders and quacks, the duty so to protect them must be found by all diligent State Boards of Health to follow sharply thereafter. This duty to repress ignorant or fraudulent practice does not, of necessity, inhere in a Board of Health--in England, for example, it rests with the General Council of Medical Education and Registration --but it has been found expedient in this country to make the State Boards the means for the correction of this form of abuse. In two States, at least, Illinois and California, a considerable improvement in medical practice has been accomplished, and we expect that West Virginia will presently report that her ranks have been purified from the worst of her quackish and ignorant impostors.--*Medical News.*

